# Dried Blood Spot Metabolome Features of Ischemic–Hypoxic Encephalopathy: A Neonatal Rat Model

**DOI:** 10.3390/ijms25168903

**Published:** 2024-08-15

**Authors:** Chupalav Eldarov, Natalia Starodubtseva, Yulia Shevtsova, Kirill Goryunov, Oleg Ionov, Vladimir Frankevich, Egor Plotnikov, Gennady Sukhikh, Dmitry Zorov, Denis Silachev

**Affiliations:** 1 V.I. Kulakov National Medical Research Center for Obstetrics Gynecology and Perinatology, Ministry of Healthcare of Russian Federation, 117997 Moscow, Russia; ch_eldarov@oparina4.ru (C.E.); n_starodubtseva@oparina4.ru (N.S.); yu_shevtsova@oparina4.ru (Y.S.); k_gorunov@oparina4.ru (K.G.); o_ionov@oparina4.ru (O.I.); v_frankevich@oparina4.ru (V.F.); plotnikov@belozersky.msu.ru (E.P.); g_sukhikh@oparina4.ru (G.S.); 2 A.N. Belozersky Institute of Physico-Chemical Biology, Lomonosov Moscow State University, 119992 Moscow, Russia; 3 Moscow Center for Advanced Studies, 123592 Moscow, Russia; 4 Laboratory of Translational Medicine, Siberian State Medical University, 634050 Tomsk, Russia

**Keywords:** metabolomics, lipidomics, diagnostics, neonatal asphyxia, liquid chromatography–mass spectrometry

## Abstract

Hypoxic–ischemic encephalopathy (HIE) is a severe neurological disorder caused by perinatal asphyxia with significant consequences. Early recognition and intervention are crucial, with therapeutic hypothermia (TH) being the primary treatment, but its efficacy depends on early initiation of treatment. Accurately assessing the HIE severity in neonatal care poses challenges, but omics approaches have made significant contribution to understanding its complex pathophysiology. Our study further explores the impact of HIE on the blood metabolome over time and investigated changes associated with hypothermia’s therapeutic effects. Using a rat model of hypoxic–ischemic brain injury, we comprehensively analyzed dried blood spot samples for fat-soluble compounds using HPLC-MS. Our research shows significant changes in the blood metabolome after HIE, with a particularly rapid recovery of lipid metabolism observed. Significant changes in lipid metabolites were observed after 3 h of HIE, including increases in ceramides, carnitines, certain fatty acids, phosphocholines, and phosphoethanolamines, while sphingomyelins and *N*-acylethanolamines (NAEs) decreased (*p* < 0.05). Furthermore, NAEs were found to be significant features in the OPLS-DA model for HIE diagnosis, with an area under the curve of 0.812. TH showed a notable association with decreased concentrations of ceramides. Enrichment analysis further corroborated these observations, showing modulation in several key metabolic pathways, including arachidonic acid oxylipin metabolism, eicosanoid metabolism via lipooxygenases, and leukotriene C4 synthesis deficiency. Our study reveals dynamic changes in the blood metabolome after HIE and the therapeutic effects of hypothermia, which improves our understanding of the pathophysiology of HIE and could lead to the development of new rapid diagnostic approaches for neonatal HIE.

## 1. Introduction

Hypoxic–ischemic encephalopathy (HIE) is a serious neurological condition associated with perinatal asphyxia at birth, leading to a wide spectrum of neurological impairments. In high-income countries with advanced medical infrastructure and obstetric care, the reported incidence of moderate-to-severe HIE ranges from about 1 to 8 cases per 1000 live births [1]. While neonatal HIE is relatively rare compared to other birth-related complications, it can have significant short- and long-term consequences for about 47% (95% CI 36 to 57) of affected infants, including death, cerebral palsy, epilepsy, seizures developmental delays, and motor and cognitive deficits [2]. Early recognition, prompt medical intervention, and appropriate supportive care are critical in improving outcomes for infants with HIE [3].

The challenges posed by these significant disabilities highlight the importance of early diagnosis and rapid intervention to mitigate the long-term effects of HIE on affected infants. Timely recognition of HIE symptoms, followed by appropriate medical interventions and comprehensive rehabilitative care, is critical for improving outcomes and enhancing the quality of life for individuals impacted by this condition. Ongoing research and advancements in medical technology play an advanced role in furthering our understanding of HIE and developing innovative approaches to diagnosis, treatment, and long-term management strategies.

Therapeutic hypothermia (TH) remains the only effective treatment for neonatal moderate-to-severe HIE, widely implemented in neonatal intensive care units (NICUs) around the world [4]. A recent meta-analysis demonstrated that TH significantly reduces the risk of death in neonates with moderate-to-severe HIE. Both selective head cooling and whole-body cooling have proven to be beneficial in reducing mortality in infants with this condition [5]. TH helps to reduce metabolic rate, decrease excitotoxicity, inhibit inflammation, and prevent apoptosis in the brain, thereby protecting neurons from further damage caused by oxygen deprivation and reduced blood flow [6]. Unfortunately, the therapeutic window for treating HIE with TH is limited to the first 6 h of life [7,8]. The rationale behind initiating therapeutic hypothermia within this early time window is to intervene before secondary injury mechanisms, such as inflammation and apoptosis, become prominent. Hypothermia helps to mitigate these secondary injury processes, preserving brain tissue and improving neurological outcomes in affected infants [9]. The efficacy of therapeutic hypothermia decreases over time, which emphasizes the importance of early recognition and immediate initiation of treatment in appropriate infants.

Early and accurate assessment of HIE severity poses a significant challenge in neonatal care [3,7,10]. Clinical studies during the neonatal period are often limited and influenced by concurrent pathologies such as sepsis or inborn errors of metabolism [11]. Several animal models have been developed to address this problem [11,12,13]. Omics approaches, including genomics, transcriptomics, proteomics, and metabolomics, have significantly contributed to unraveling the complex pathophysiological mechanisms underlying HIE: alterations in energy metabolism, oxidative stress, neurotransmitter and hormone metabolism, and other metabolic pathways implicated in neuronal injury and neuroprotection [7,11,14]. Recent systematic reviews have identified significant alterations in alanine, aspartate, and glutamate metabolism as key pathways affected by HIE [3,15]. Glutamate, a pivotal neurotransmitter, plays a crucial role in acute neurological conditions, including HIE [3,15].

The pathogenesis of HIE comprises four distinct phases: the acute (0–6 h), the short “latent”, the secondary (up to 3 days), and the tertiary phase, each characterized by specific molecular patterns and cellular events [11]. Longitudinal studies have revealed remarkable changes in amino acid content, particularly of glycine and methionine, as well as in acylcarnitines, succinylacetone, choline, 6,8-dihydroxypurine, and hypoxanthine, during the course of HIE [16,17]. Preferably, non-invasive or minimally invasive samples, such as dried blood spots (DBS), should be used in these studies, especially for dynamic assessments. Overall, metabolomics is a promising tool to improve our understanding of HIE pathophysiology, increase diagnostic and prognostic potential, guide therapeutic interventions, and ultimately improve outcomes for infants affected by this devastating neurological disorder.

In this study, we further investigated the effects of HIE on the blood metabolome over time and explored the changes associated with the therapeutic effects of hypothermia. The molecular composition of DBS samples obtained from a recent study using the modified Rice–Vannucci model of hypoxic–ischemic (HI) brain injury in 7-day-old rats [16] was comprehensively analyzed using non-targeted shotgun analysis of fat-soluble compounds (HPLC-MS).

## 2. Results

**Figure 1:**
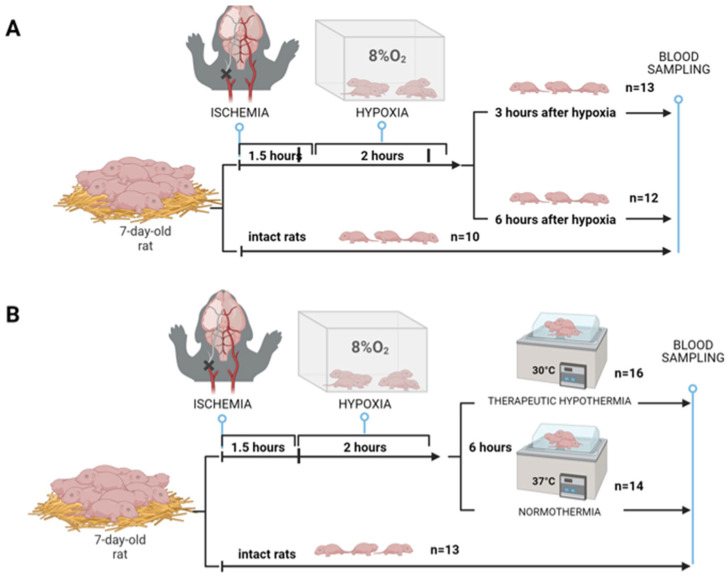
Study design: (**A**) HIE with sampling at different times; (**B**) modeling of therapeutic hypothermia [16].

To identify specific markers of HI-related brain damage and assess metabolic changes in the blood during TH, all samples were derived in the form of dried blood spots from the two previous series of experiments, as described in the study by Shevtsova and co-authors [16] (Figure 1). The primary aim was to investigate the temporal shifts in blood metabolism: before the onset of HIE and 3 and 6 h after HIE. A second series of experiments aimed to clarify the effects of hypothermia on the molecular composition of the blood.

### 2.1. Time-Related Features of Metabolome DBS Caused by Hypoxia–Ischemia

**Figure 2:**
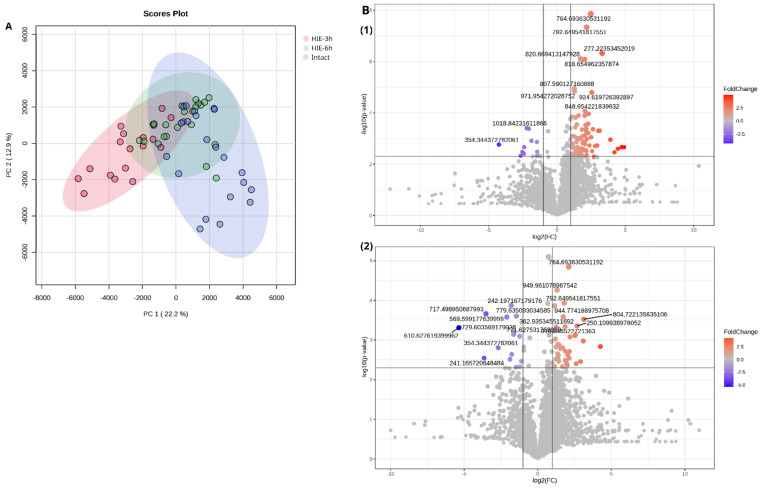
The dependence of time after HI exposure on the low-molecular-weight spectrum of DBS: (**A**) Principal component analysis (PCA) space based on blood compounds, whose levels show statistically significant differences in pairwise group comparisons; (**B**) Volcano plots of DBS metabolites levels over time: (**1**) 3 h after HIE, (**2**) 6 h after HIE.

Partial least squares discriminant analysis (PLS-DA) of the three groups shows that the control group samples and the samples collected 3 h after HI are distinctly separated from each other, while the 6 h samples occupy an intermediate position (Figure S1). Figure 2A presents the results of principal component analysis (PCA) for blood compounds, whose levels show statistically significant differences in pairwise group comparisons. PCA plot follows the same pattern, with the 3 h group being more distinct from the control than the 6 h group. More than half of the lipids detected in rats DBS demonstrated statistically significant (*p* < 0.05) correlations with each other (Figure S2). This finding confirms that the alterations in blood lipid profiles induced by HIE are interconnected through specific molecular pathways.

- *3 h after HIE*

**Figure 3:**
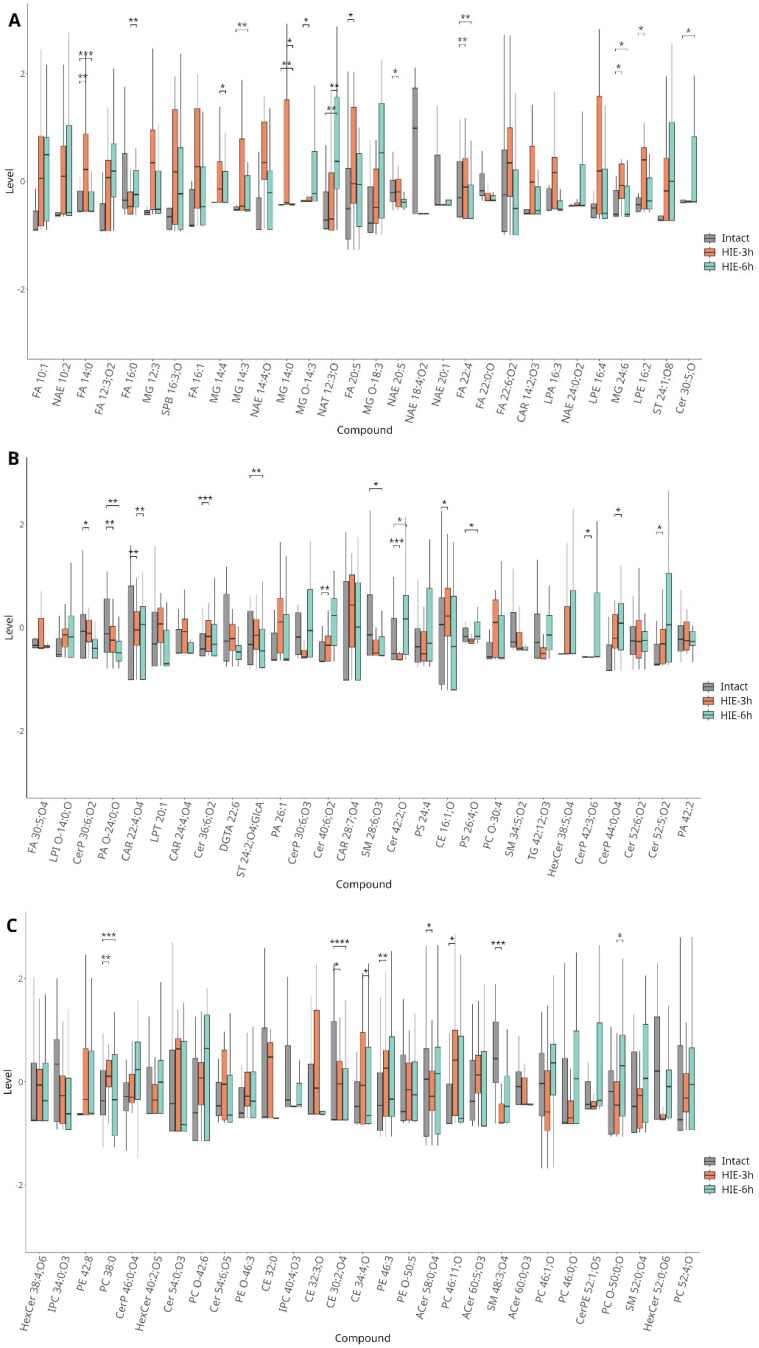
Box plots representing the concentration levels of metabolites that show significant changes over time. Plots are divided to three subgroups (**A**–**C**) to improve readability. Data are presented as mean  ±  SD. * *p* < 0.05, ** *p* < 0.01, *** *p* < 0.001, **** *p* < 0.0001.

Three hours after hypoxia, a statistically significant (*p* < 0.01) increase in the levels of phosphocholines (PCs), phosphoethanolamines (PEs), ceramides (Cers), carnitines (CARs), and free fatty acids (FAs) and short-chain *N*-acylethanolamines (NAEs) was observed, while sphingomyelins (SMs) and long-chain NAEs decreased (Figure 3).

- *6 h after HIE*

**Figure 4:**
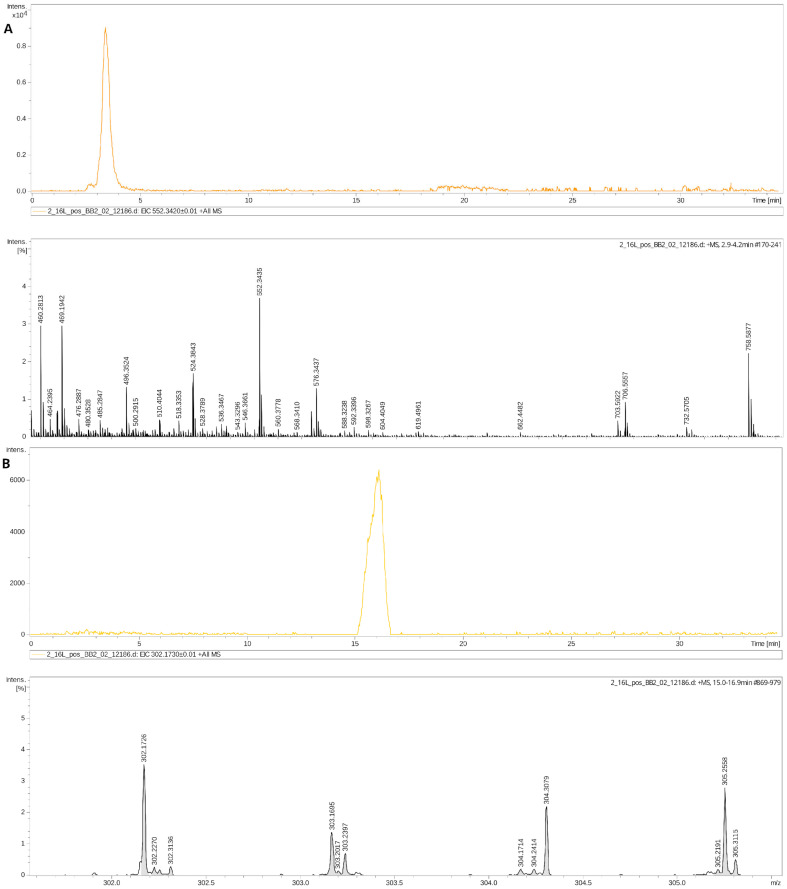
Examples of chromatograms and mass spectrum of metabolites: (**A**) Cer P30:6;O2 (*m*/*z* 552.342); (**B**) NAE 14:4;O (*m*/*z* 302.173).

Six hours after induced injury, changes in the DBS metabolome were primarily associated with the same classes as at three hours, including Cers, FA, and phospholipid (PL) species (Figure 3). However, the overall number of changed patterns was slightly smaller than at 3 h and the fold change was also lower (Figure 2B). This suggests a rapid normalization of blood lipid profiles following the initial perturbation caused by hypoxic–ischemic events. The complete list of annotated compounds specific for HIE with abbreviations is added to Supplementary Tables S1 and S2. Figure 4 shows the examples of chromatograms and mass spectrum of metabolites with the pronounced disturbance via HI influence: CerP 30:6;O2 (*m*/*z* 552.342, FC = 2.5, *p* = 0.0002) and NAE 14:4;O (*m*/*z* 302.173, FC = 2.5, *p* = 0.0004).

### 2.2. Therapeutic Effect of Hypothermia in Hypoxic–Ischemic Injury

**Figure 5:**
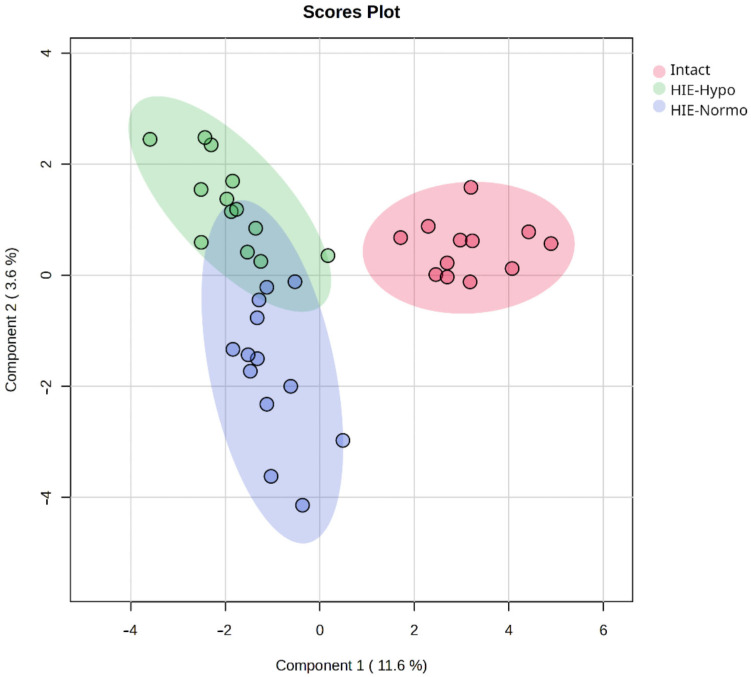
PLS-DA score plot of DBS samples after hypoxia and therapeutic hypothermia compared to normothermia and control group.

In the second set of experiments, one-third of the rats subjected to hypoxia–ischemia were subsequently exposed to 6-h hypothermia (Figure 1B). This treatment induced significant changes in the blood metabolome of the rats when compared to the normothermic conditions (Figure S3). Despite these substantial metabolic shifts, the hypothermia did not fully reverse the pathological processes caused by hypoxia–ischemia (Figure 5). After HI followed by hypothermia, there was a statistically significant decrease in the levels of ceramides, sphingomyelins, and triglycerides compared to the normothermic group after HI, while fatty acids were upregulated (Table S3). Enrichment analysis indicated Arachidonic acid oxylipin metabolism, Eicosanoid metabolism via lipooxygenases (LOX), and Leukotriene C4 Synthesis Deficiency pathways as the most significant based on the identification of molecular patterns (Figure S4).

### 2.3. Development of the Diagnostic Model for HIE

**Figure 6:**
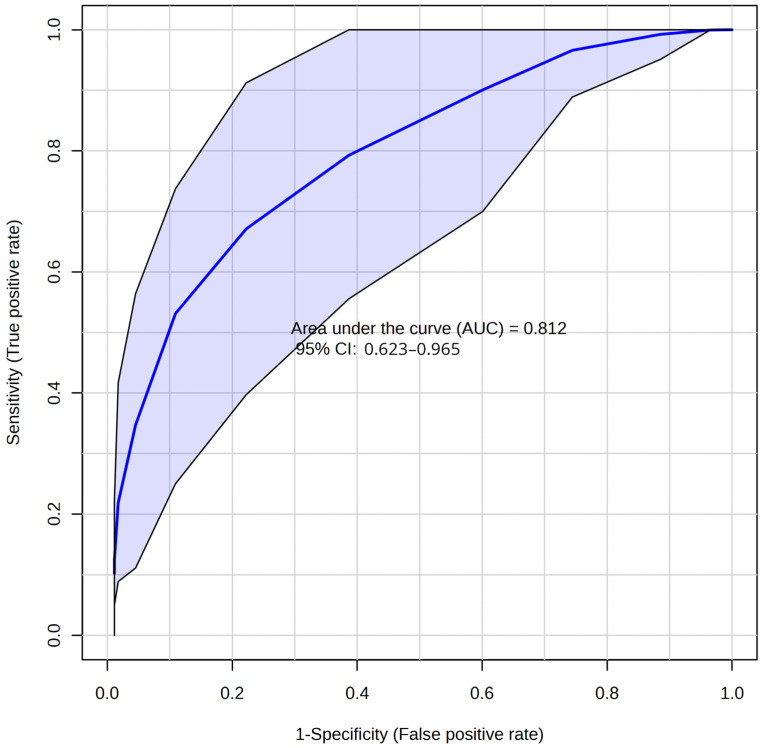
ROC curve for diagnosing after 3 h post-HIE based on OPLS-DA model.

Additionally, for the diagnosis of HI based on the DBS profile 3 h after the presumed event, an orthogonal partial least squares discriminant analysis (OPLS-DA) model with two orthogonal projections was created for both the 3 h and 6 h after HIE groups. According to receiver operating characteristic (ROC) curves, the model based on the HIE-3h DBS metabolome showed better diagnostic performance (AUC 0.812 vs. 0.767) than the HIE-6h (Figure 6 and Figure S5). The most significant features included fatty acids, *N*-acylethanolamines, and *N*-acyl-taurines.

## 3. Discussion

HIE is a major challenge due to the limitations of current clinical tools and procedures [18,19,20]. Although methods such as amplitude-integrated EEG (aEEG) and MRI are widely used to assess the severity of brain injury and predict long-term outcomes in HIE patients, their efficacy is often insufficient in the crucial early stages of life when rapid and accurate diagnosis is essential for deciding on TH as a treatment option [20,21,22]. A major limitation of these instruments is their timing and sensitivity. While aEEG is invaluable for continuous monitoring, it cannot always detect subtle or emerging abnormalities immediately after birth, potentially delaying intervention. MRI, while very detailed, is often not feasible in the immediate postnatal period and may require sedation, which is not good for critically ill neonates. These delays and limitations compromise the ability to initiate TH within the narrow therapeutic window, which is typically within the first six hours after birth and is critical for improving neurological outcomes [21,22,23].

To overcome these limitations, several potential solutions and future research directions could be considered [21,22,23,24,25]. First, use of metabolomics and proteomics could lead to the identification and validation of specific biomarkers in blood or urine that indicate early neuronal damage. These markers could provide a faster and non-invasive way to diagnose HIE and provide valuable diagnostic information even in cases where traditional imaging is still inconclusive. Second, improvements in aEEG technology, including the development of higher resolution and higher sensitivity devices, could allow for better early detection of subtle changes in brain activity. Coupling aEEG with advanced algorithms for automatic pattern recognition could increase the reliability and speed of diagnosis. Third, innovations in portable MRI technology and the development of rapid, neonatal-specific imaging protocols could allow brain scans to be performed in NICUs shortly after birth, reducing the need for transportation and sedation. In addition, the combination of aEEG, near-infrared spectroscopy (NIRS), and other physiologic monitoring techniques could provide a more comprehensive overview of cerebral oxygenation and hemodynamics in real-time, aiding in the early identification of infants at risk for HIE. These advances would allow healthcare professionals to immediately recognize infants at high risk of HIE and take timely and effective interventions that have the potential to significantly improve treatment outcomes and long-term prognoses for these vulnerable patients.

Furthermore, medicine faces a major challenge in the diagnosis of HIE due to the wide-ranging effects of hypoxia on various organs and tissues. These wide-ranging effects can profoundly alter the molecular profile of the samples studied, making the identification of HIE markers difficult. Understanding the impact of hypoxia on various molecular processes is essential for the accurate diagnosis and treatment of HIE. To address this issue, researchers often use model experiments to create highly controlled conditions. These experiments facilitate the investigation of potential molecular markers associated with specific responses to different stimuli, particularly those related to hypoxic–ischemic conditions [26,27,28]. Experimental models allow researchers to more effectively isolate and identify molecular markers indicative of HI damage. In the field of HIE research, in vivo models of HI brain injury in animal studies have proven to be valuable tools for studying the effects of hypoxia on molecular processes. These models, commonly used in studies with rats, mice, and primates, provide insight into the molecular changes that occur in response to HI insult [29,30]. Using these models, researchers can gain a deeper insight of the molecular mechanisms underlying HIE and identify potential markers for diagnosis and treatment. In our studies, we focused on the well-established Rice–Vannucci model of neonatal HI to mimic brain injury [14,16]. In this model, both systemic hypoxia and cerebral ischemia are induced by unilateral transection of the carotid artery [31,32]. Using this model, we aimed to gain a more comprehensive understanding of the molecular responses to the HI insult and identify potential diagnostic markers for HIE.

The use of DBS in neonatal biomarker research for HIE offers numerous advantages. DBS has been used in neonatal metabolic screening programs for more than five decades [33,34]. The samples can be conveniently stored for long time without the need for elaborate preservation methods, making them suitable for longitudinal studies or retrospective analysis [35]. In addition, the application of advanced “omics” technologies to analyze metabolites in DBS samples holds great potential for the identification of new biomarkers associated with HIE. Techniques such as metabolomics and proteomics facilitate the comprehensive profiling of biochemical compounds in the blood and provide insights into the metabolic dysregulations underlying the pathophysiology of HIE [36,37]. By utilizing the rich metabolic information in DBS samples, researchers can identify specific biomarkers that reflect disease severity, prognosis, or response to treatment. This integrated approach enables the discovery of new biomarkers and contributes to the development of precision medicine strategies tailored to the metabolic profiles and treatment needs of individual patients [38].

In this study, PLS-DA analysis of chromatography–mass spectrometry data of organic extracts from DBS collected 3 and 6 h after induced HIE revealed a distinct separation between the experimental groups. The early changes in blood lipid profiles (3 h) were more pronounced compared to the later time points (6 h). Therefore, early sampling allows a more accurate diagnosis of HI based on lipid profiles. Interestingly, amino acids and acylcarnitines showed less prominent changes in the blood molecular spectrum within the investigated time interval [16].

A metabolomic analysis of DBS demonstrated the significant involvement of lipids in the pathophysiology of perinatal asphyxia. A statistically significant (*p* < 0.05) increase of more than 2-fold was observed in the levels of phosphatidylcholines (PCs), phosphatidylethanolamines (PEs), monoglycerides (MGs), diglycerides (DGs), phosphatidylserines (PSs), lysophosphatidylethanolamines (LysoPEs), phosphatidic acids (PAs), ceramides (Cers), and acylcarnitines (CARs), together with a decrease in long-chain *N*-acylethanolamines (NAEs) and multidirectional changes in fatty acids, sphingomyelins (SMs), and triacylglycerides (TGs) in DBS during the development of HI injury. Previous studies have identified choline as the most significantly disturbed lipid metabolite in HIE, with levels increasing more than eightfold [39,40,41]. Furthermore, the research team led by Kuligowski developed a comprehensive metabolite score that includes choline, 6,8-dihydroxypurine, and hypoxanthine. This metabolite score demonstrated a strong correlation with the duration of hypoxia in a piglet model of HIE [17]. Choline and its derivatives play crucial roles in maintaining the structural integrity of cell membranes as phospholipids, facilitating neurotransmission via acetylcholine synthesis, supporting lipid transport through lipoproteins, and managing methyl group metabolism by reducing homocysteine levels [42].

During neonatal HI conditions, there is an excessive release of glutamate, resulting in excitotoxicity that damages neuronal cells. This increase in glutamate increases the influx of calcium ions into the neurons, leading to calcium overload and further exacerbating cellular damage. At the same time, the production of reactive oxygen species (ROS) is increased further stressing the neuronal environment. These biochemical cascades cumulatively contribute to significant neuronal damage and potentially cell death [43]. Elevated intracellular Ca^2+^ levels activate membrane phospholipases (PLA2s), proteases, and nucleases [44], leading to the hydrolysis of membrane phospholipids by PLA2s and the release of free fatty acids (FA), lysophosphatidylcholines, diacylglycerides, eicosanoids, and lipid peroxides [45]. In this experimental study, significant changes were observed in fatty acids (FA 10:1, 12:3, 14:0, 16:0, 16:1, 20:5, 22:0, and 22:4), lysophosphatidylethanolamines (lysoPE(16:2) and lysoPE(20:5)) in DBS, which is consistent with the results of another study [46]. Lysophosphatidylcholines serve as potent mediators of brain inflammation by stimulating interleukin-1β release and subsequent microglial activation [47,48]. A piglet model study by Solberg R, et al. revealed a more than sixfold increase in blood FA levels as well [40].

Brain tissue is particularly vulnerable to oxidative stress due to its high metabolic rate and the polyunsaturated fatty acid-rich composition of the neuronal membrane. In addition, the brain has a lower antioxidant content than other organs [44]. The activity of PLA2, which can yield arachidonic acid (AA) that is subsequently converted into pro-inflammatory compounds such as prostaglandins, significantly exacerbates oxidative damage in neural tissue after hypoxia and reoxygenation [49,50,51]. At the end of the acute phase and at the beginning of the latent period of hypoxic–ischemic encephalopathy (HIE), a significant increase in eicosapentaenoic acid (EPA, ω-3 FA 20:5) was observed. EPA, a precursor of docosahexaenoic acid (DHA), and both EPA- and DHA-derived eicosanoids decrease inflammation [52].

Furthermore, previous studies have reported an increase in AA (FA 20:4) and linoleic acid (LA, FA 18:2) levels in response to HI injury while docosatetraenoic acid (FA 22:4) significantly decreased (FC = −3.13, *p* < 0.001) in blood 6 h after induced damage [14]. FA 22:4, which is derived from the ω-6 polyunsaturated fatty acid AA, is one of the predominant fatty acids in the early development of the human brain [53,54]. The decreased FA 22:4 levels during the latent phase of HI brain injury may reflect the increased metabolism of this fatty acid in response to increased levels of ROS, the development of an immune-inflammatory cascade, and the onset of mitochondrial dysfunction in brain cells [54].

Thus, in response to HI injury, there are significant changes in the fatty acid components of brain lipids, including AA, EPA, LA, and adrenic acids. Furthermore, the direction and effects of these changes vary, potentially leading to interrelated (both amplifying and attenuating) cascades of molecular processes at the systemic level.

Moreover, during the latent phase of HIE, the brain suffers from acute glucose deficiency, its primary energy source. To compensate for these energy deficits, beta-oxidation of lipids in the mitochondria is activated. Consistently, a statistically significant increase in phospholipids and triglycerides degradation products—monoglycerides (particularly, MG 14:0 increased 30-fold, *p* < 0.01), diglycerides (DG28:4;O, DG48:0;O), phosphatidic acids (PA 26:1, PA 42:2, PA O-24:0;O), and fatty acid transporters through the mitochondrial membrane—acylcarnitines (CAR14:2;O3, CAR20:4, CAR22:4;O4, CAR28:7;O4), was observed. The positive correlation between acylcarnitine levels and time elapsed after the traumatic event in this study is consistent with our previously published research [14]. The most significant changes in DBS collected 3 h after HI were observed for arachidonoylcarnitine (CAR 20:4; FC = 4.3, *p* < 0.01).

Acylcarnitines are formed by the esterification of carnitine, which is necessary for the transport of fatty acids across the mitochondrial membrane for subsequent beta-oxidation and energy production. Under hypoxia–ischemia, cellular energy production is impaired, leading to metabolic changes and a shift in bioenergetics toward anaerobic glycolysis. This affects the levels of various metabolites, including acylcarnitines [40,55]. The accumulation of certain acylcarnitines may indicate mitochondrial dysfunction and impaired energy metabolism. Clinical studies have shown that changes in the acylcarnitine profile can serve as biomarkers for HIE. For example, increased concentrations of long-chain acylcarnitines were found in the umbilical cord blood of newborns with HIE [56]. In a rat model subjected to hypoxic conditions, administering L-carnitine notably decreased the levels of lactate in the brain as well as the byproducts of lipid peroxidation [57]. This finding suggests that carnitines may act as protective mechanisms, particularly in highly oxygen-dependent tissues, when faced with hypoxia. The study highlights the potential role of L-carnitine in mitigating the biochemical impacts of oxygen deprivation, underscoring its therapeutic value in enhancing tissue resilience against hypoxic insult.

In summary, although changes in acylcarnitines were initially statistically significant, these species were excluded as reliable biomarkers for the diagnosis of HIE after applying the false discovery rate (FDR) correction. Since the source of acylcarnitines is predominantly skeletal muscle as a result of systemic HI events, the early increase in acylcarnitine levels after HIE might reflect systemic hypoxia rather than indicate the extent of cerebral injury. Therefore, acylcarnitines could serve as markers of systemic hypoxia in neonates and indirectly indicate possible brain damage, the organ most susceptible to oxygen deficiency. Nevertheless, comprehensive, long-term, multicenter studies of changes in acylcarnitine levels in neonates with HIE are needed to draw definitive conclusions.

*N*-Acylethanolamines (NAEs) are fatty acid amides that play a critical role in inflammation, neurotransmission, and stress. The messenger function of NAEs is modulated by the specific type of fatty acyl group they contain. Previous research has shown that NAEs accumulate in the brain following injury and exert neuroprotective effects through several mechanisms [58,59]. The neuroprotective function of NAEs is thought to be related to an increase in ceramide levels, inhibition of mast cell activation, and degradation of anandamide [59]. Studies have shown that stearoyl-ethanolamine can alleviate LPS-induced brain damage in mice [60]. In our study, we observed an increase in short-chain NAE levels 3 h after hypoxia, confirming recent findings and emphasizing the importance of NAEs in the damage control system of the brain.

Another group of lipids that undergo remarkable changes after HIE are the ceramides as well as their derivatives and sphingomyelins as precursors. As mentioned above, NAEs exert neuroprotective functions by increasing ceramide levels. While ceramides have traditionally been considered stable components of membranes, it is now apparent that they have a strong signaling and regulatory role in various cellular processes, including proliferation, apoptosis, and senescence [61]. Elevated ceramide levels resulting from the hydrolysis of sphingomyelin contribute to HIE-induced brain damage and trigger mitochondria-dependent apoptosis activation [62]. Ceramide aberrations and resulting sphingolipid-associated pathologies can lead to a variety of disorders, such as epilepsy, leukodystrophy, Alzheimer’s disease, and even congenital ichthyosis, a severe skin disease [63]. The diverse spectrum of possible pathological consequences of impaired ceramide metabolism emphasizes its importance for normal physiology. Several studies demonstrated changes in ceramide levels after acute ischemic stroke (AIS), and ceramide levels have been proposed as a prognostic marker for assessing the outcome of AIS [64,65,66]. In our study, we observed significant changes in a variety of ceramides, including acyl-ceramides and ceramide-phosphates, 3 h after HI, reflecting the brain damage that had occurred.

In a landmark study conducted by Gunn and colleagues on fetal sheep in 1998, it was shown that lowering the head temperature before the onset of ischemic symptoms and maintaining this temperature for 3 days can significantly improve the outcome of severe HIE [67]. Later research confirmed the positive effects of hypothermia, as a review and meta-analysis of seven studies showed, indicating a reduction in mortality and an increase in survival in infants with moderate-to-severe encephalopathy [68]. As a result, therapeutic hypothermia has become the standard treatment for infants after 36 weeks of gestation with moderate or severe HIE. Despite the extensive clinical application of this method over many years, the exact effect of hypothermia on neonatal metabolism remains largely unclear, as very few specific metabolomic studies have been published to date. In our study, we observed a decrease in *N*-acylethanolamines (NAEs) and ceramides in the hypothermia group compared to the normothermia group, suggesting a potential reduction in the severity of HIE symptoms and the subsequent organismal response. Our results reveal a significant shift in lipid metabolism during therapeutic hypothermia, which likely contributes to its positive effects on the treatment of HIE. It is essential to recognize that numerous studies have consistently underscored that even after 48 h of intensive care with HT, newborns who have been successfully treated for HIE continue to exhibit distinct metabolic profiles compared to their healthy peers [69,70]. One notable change is in glutamine levels, which reflects its role as a precursor to glutamate, the primary excitatory neurotransmitter involved in perinatal brain injury [71]. Additionally, hypoxanthine levels are frequently highlighted in these studies due to their clinical significance. Hypoxanthine interacts with xanthine oxidase to promote the formation of harmful ROS, and an increase in hypoxanthine levels is often considered a key biochemical indicator of asphyxia [72].

As a limitation of our study, it is important to mention that the animals were exposed to systemic hypoxia, which could have affected other organs and tissues, possibly altering the metabolic profile studied, such as the carnitine levels discussed above. Secondly, semi-quantitative lipidomic analysis does not allow for the precise identification of substances corresponding to potential marker ions (*m*/*z*). In the future, we plan to perform a targeted lipidomic analysis with the selection of appropriate MRM and internal standards.

## 4. Materials and Methods

In this study, all investigations were performed with dried blood spot samples taken from a previous study described in detail by Shevtsova et al. [16]. In the previous study, a sufficient amount of dried blood spots remained from each experimental animal to perform a separate analysis of lipid markers of brain damage. The use of samples from previous animal studies is consistent with the 3R principles, which promote ethical research practices by reducing the need for additional animal testing.

### 4.1. Rice–Vannucci HIE Rat Model

In this study, the principles of the 3Rs were strictly adhered to to ensure ethical treatment of the laboratory animals. The animal protocols used in this study were thoroughly reviewed and approved by the institutional animal ethics committee in accordance with the guidelines of FELASA (Federation of European Laboratory Animal Science Associations). For the experiments, outbred white rats obtained from the animal facility of the A.N. Belozersky Institute of Physico-Chemical Biology were used. To ensure the welfare of the animals, the dams and their pups were housed in cages in a temperature-controlled environment at a temperature of 21 ± 2 °C. A light/dark cycle was also used. In addition, a light/dark cycle was set up with the lights on from 9:00 to 21:00. Pregnant female rats were observed daily to monitor their condition and ensure appropriate care. After the pups were born, their condition was carefully monitored for one week, including visual inspection and confirmation of milk spotting in the stomach projection.

In the study, the widely recognized Rice–Vannucci rat model was used to investigate HIE. Seven-day-old postnatal rats of both sexes were selected for the experiments. Pups were anesthetized with 1% isoflurane administered via a low-flow anesthesia machine (SomnoSuite, Kent Scientific, Torrington, CT, USA). The left carotid artery was then surgically isolated and electrocauterized. Pups were exposed to a gas mixture of 8% oxygen and 92% nitrogen at 37 °C in a multigas CO_2_ incubator (Binder, Tuttlingen, Germany). Exposure lasted 2 h and started 1.5 h after surgery. After surgery, the pups were carefully monitored hourly to detect seizures and other signs of pain or complications. The mortality rate after HI induction was between 3 and 5%.

### 4.2. HIE with Sampling at Different Times

Rats were randomly assigned to the following experimental groups: intact rats (Intact, *n* = 10), animals with HIE whose blood samples were collected after 3 h (HIE-3h, *n* = 13), and animals with HIE whose blood samples were collected after 6 h (HIE-6h, *n* = 12) (Figure 1A). Dry blood spots were obtained three and six hours after exposure to HI.

### 4.3. Modeling of Therapeutic Hypothermia

Rats were randomized to either normothermic or hypothermic recovery immediately after termination of hypoxia over a period of 6 h. Normothermic recovery was performed at a temperature of 37 °C, while hypothermic recovery was performed at 30 °C. Pups were placed in an open container in a water bath to achieve the desired temperatures. In this experimental setup, there were three groups of animals: intact rats (Intact, *n* = 13), rats with HIE and hypothermia (HIE-Hypo, *n* = 16), and rats with HIE and normothermia (HIE-Normo, *n* = 14) (Figure 1B). Similar to previous experiments, DBS were collected after 6 h of hypoxic exposure for further analysis.

### 4.4. DBS Metabolome Analysis (HPLC-MS)

Metabolites were extracted from dry blood spots using the Folch method according to the appropriate protocol [61]. Each sample received 480 µL of a chloroform–methanol mixture (2:1 ratio) and 250 µL of water. The extraction mixture was shaken vigorously for 10 min and then centrifuged at 15,000× *g* for 10 min. The organic phase (150 mL) that was collected at the bottom was transferred to a separate tube. This extraction process was repeated with the addition of another chloroform–methanol mixture. The supernatant from the second organic phase was combined with the previously collected one. The resulting mixture was dried with nitrogen at room temperature and then redissolved in 100 mL of an acetonitrile–isopropanol mixture (1:1 ratio).

Subsequent analysis of the organic phase was conducted using high-performance liquid chromatography coupled with mass spectrometry (HPLC-MS). We used an Atlantis T3 C18 column (3 µm, 15 cm in length, with an inner diameter of 1 mm, Waters, Milford, MA, USA) and the Ultimate 3000 Nano LC chromatography system (Thermo Scientific, Waltham, MA, USA) [58,59]. Reverse-phase chromatography was employed, with mobile phase “A” consisting of a mixture of ACN:H20 (60:40) and mobile phase “B” consisting of IPA:ACN:H_2_O (90:8:2). Both phases contained modifiers (0.1% formic acid and 5 mm ammonium formate). Elution was performed with a gradient of mobile phase “B” at a flow rate of 40 µL/min: 0–0.5 min at 10%, followed by a 20 min gradient from 10% to 99%. A 10 min wash with 99% of phase “B” was then performed, after which the phase was brought back to its initial concentration (10% of phase “B”) within 1 min and the column was allowed to equilibrate for 3 min. The total chromatography time for each sample was 34.5 min at a 40 um/min flow rate and column temperature of 50 °C.

Metabolites were detected using a hybrid quadrupole time-of-flight mass spectrometer, the Bruker MaXis Impact (Bruker Daltoniks, Bremen, Germany), with two replicates per sample using following instrument parameters: capillary voltage 4500 V; nebulizer pressure 0.6 Pa, dry gas flow rate 5.0 L/min with t 200 °C. The mass spectra were acquired with a resolution of 50,000 in the range of 50–1700 *m*/*z* in positive polarity. HPLC-MRM-MS was then performed to confirm the initial identification by *m*/*z* with a collision energy of 35 eV and a mass window of 2 Da.

Peak detection, grouping, and retention time correction were performed using the xcms software package(v 2.0.8). The Centwave algorithm was utilized for peak detection with specific parameters: a maximum *m*/*z* deviation of 15 ppm and a minimum-to-maximum peak width of 10 or 45 s. Peak grouping of all samples was performed using the peak density method with default parameters [59,60]. Metabolite identification was based on the LIPID MAPS database, which provides molecular weight matches (https://www.lipidmaps.org/ (accessed 3 March 2024)). MS2 spectra were analyzed using mmass software, (v. 5.5.0) followed by identification using the ms2 spectra catalog of the Human Metabolome Database (https://www.hmdb.ca (accessed 8 April 2024)).

## 5. Conclusions

Our research has elucidated notable alterations in the DBS metabolome following HIE, including a more than twofold increase (*p* < 0.05) of ceramides, carnitines, and free fatty acids and a decrease in sphingomyelins and long-chain *N*-acylethanolamines. Within three hours post-hypoxia, we observed a substantial surge in NAE and Cer species, compounds well documented to accumulate in response to neuronal injury. Notably, NAE 20:1 exhibited a dramatic increase after HIE injury (fold change = 19.1, *p* = 0.002), then slowly returned to baseline values in post-reoxygenation period (fold change = 6.45, *p* = 0.002). Moreover, NAEs were among the main features included in the OPLS-DA model for HIE diagnosis (AUC 0.812).

Interestingly, in contrast to earlier studies that noted persistent amino acid disruptions, our data indicates a rapid normalization of lipid metabolism. Significantly fewer metabolites showed marked changes six hours post-HIE compared to the control group, suggesting an asynchronous or faster recovery in lipid versus amino acid metabolism following HIE.

Furthermore, TH treatment reverses HIE-induced changes in blood lipidome. There was a statistically significant reduction in lipid classes associated with the brain damage response, such as Cer and SM, concurrent with an elevation in free fatty acid concentrations. Enrichment analysis corroborated these findings, highlighting eicosanoid metabolism as the most affected metabolic pathway. Consequently, we hypothesize that therapeutic hypothermia mitigates HIE-induced brain damage and modulates the extent of the damage response, particularly through alterations in fatty acid metabolism and eicosanoid biosynthesis.

Overall, the search for lipid markers of neuronal damage by metabolomic analysis can be assumed to be the effective and appropriate vector for early diagnosis of HIE. This study highlights the pivotal role that lipid metabolism plays in the condition, providing new insights into potential biomarkers and therapeutic targets for early diagnosis and intervention in affected neonates. The findings underscore the importance of lipids not only in the pathogenesis but also in the overall metabolic response to perinatal asphyxia, paving the way for more sophisticated and effective clinical approaches to managing and mitigating the adverse effects associated with this condition.

Although our study was primarily conducted in a rat model, the implications for clinical translation to human neonates are important. Our research demonstrates that mass spectrometric analysis of dried blood spots for lipid markers can be helpful in the identification of neonatal brain injury. Furthermore, our results show that the optimal time window for the detection of lipid markers associated with HIE is three hours after injury/birth. Future studies should focus on quantitatively analyzing the lipidomic markers we identified and their correlation with the severity of clinical outcomes in newborns.

### Statistical Methods

No specific statistical methods were used to predetermine sample sizes (*n*) both in post-HIE time-lapse and TH effect studies.

The statistical significance of differences in the relative concentrations (average integrated peak areas) of specific metabolites between groups was assessed using the t-test, with a *p*-value of less than 0.05 considered statistically significant. As an additional criterion for the identification of potential biomarkers, we applied a threshold value of at least 1.5 for the fold change.

Pathway enrichment analysis was conducted using the overrepresentation analysis of the MetaboAnalyst 6.0.0 package.

Classification models based on OPLS-DA were created for HIE diagnosis. Sensitivity and specificity were determined using the results of leave-one-out cross-validation.

A multivariate statistical analysis using PLS-DA and OPLS-DA was performed to identify and visualize the differences between the groups using the MetaboAnalyst 6.0.0 package (https://metaboanalyst.ca (accessed 16 April 2024)) both for time-lapse and TH effect study. As part of the data preprocessing, near-constant features were filtered off based on standard deviation, and Pareto scaling was applied. The resulting models were then validated using built-in permutation analysis (100×), which showed statistically significant *p*-values (<0.05) for all models built.

The following measures were taken to minimize potential sources of bias. Each litter of pups was randomly assigned to experimental groups using Microsoft Excel 16 for randomization to ensure an even distribution of confounding factors. Data analysis was performed in a blind fashion, with researchers unaware of group assignment until the bioinformatics phase to minimize subjective influence on data interpretation.

## Data Availability

Data are contained within the article and Supplementary Materials.

## Supplementary Materials

The supporting information can be downloaded at: https://www.mdpi.com/article/10.3390/ijms25168903/s1.
