# Supplementary material for: Dried Blood Spot Metabolome Features of Ischemic–Hypoxic Encephalopathy: A Neonatal Rat Model
Source: Int J Mol Sci. 2024 Aug 15;25(16):8903. doi: 10.3390/ijms25168903 (PMC11354919; doi:10.3390/ijms25168903)
Supplement: Supplementary file 1 [file ijms-25-08903-s001.zip › ijms-3055202-supplementary.pdf]

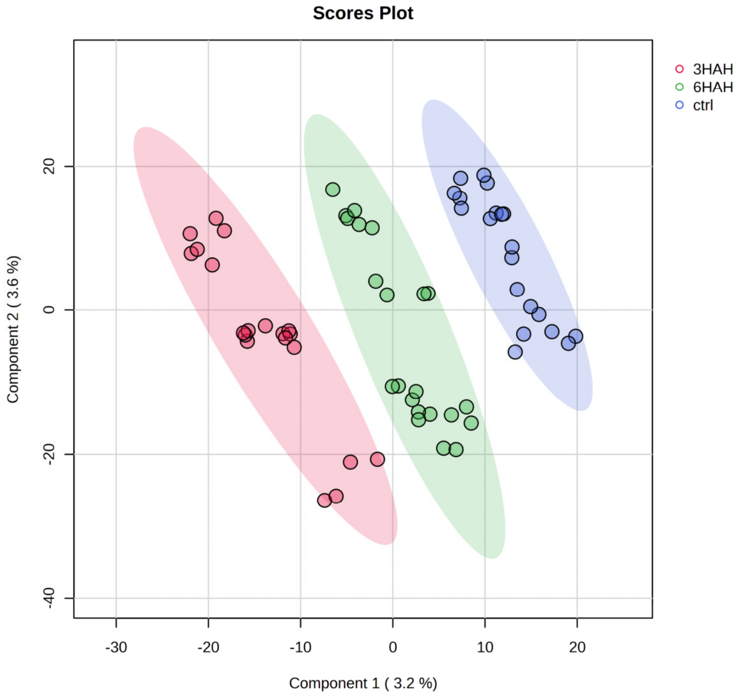

Figure S1. The impact of time after hypoxic-ischemic exposure on the low-molecular-weight spectrum of DBS: A) Sparse Partial Least Squares Discriminant Analysis (sPLS-DA) based on blood compounds, whose levels show statistically significant differences in pairwise group comparisons; B) Correlation diagram of statistically significant compounds associated with the time elapsed since HIE.

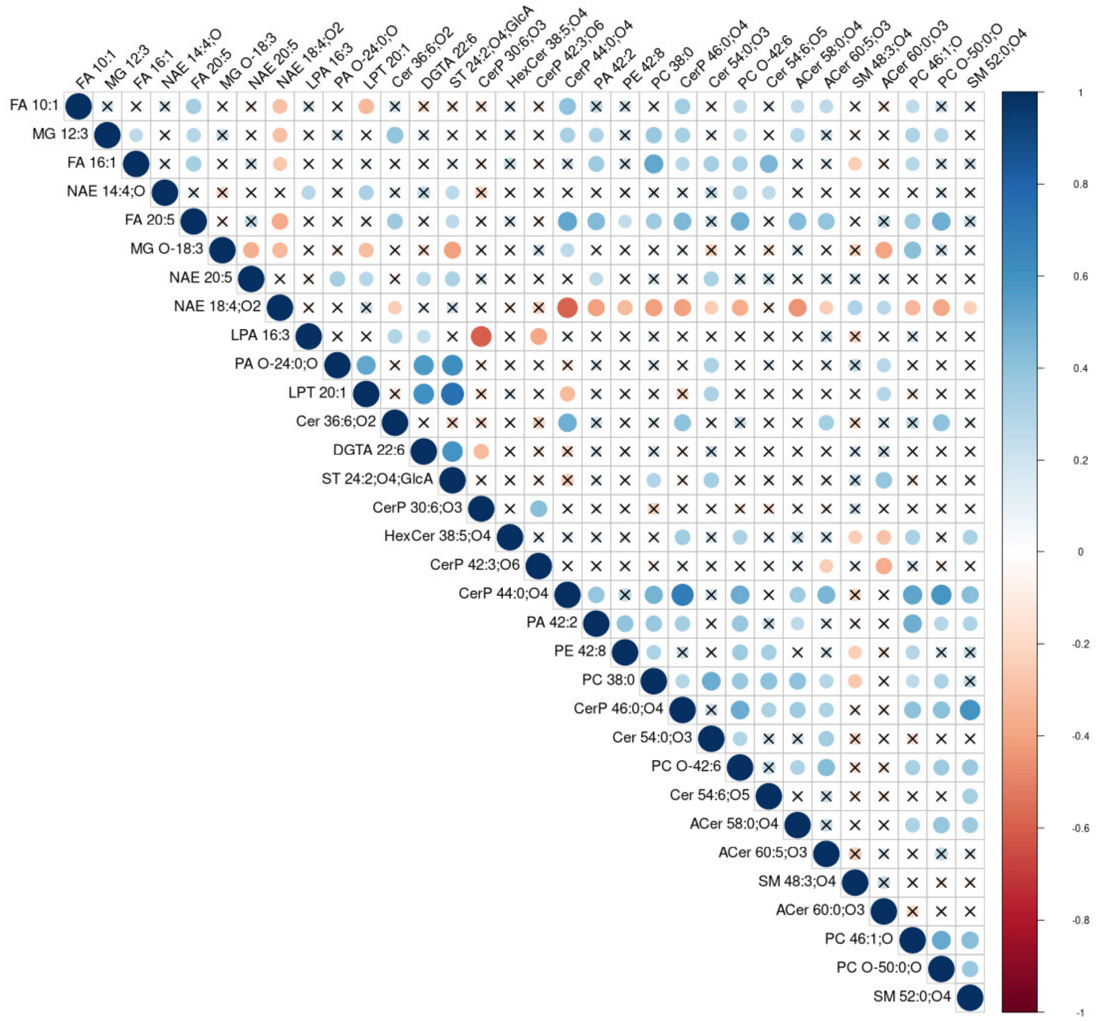

Figure S2. Correlation diagram of the compounds that show statistically significant associations X is a label indicating the absence of a significant association.

Table S1. Possible biomarkers with statistically significant changes in concentration at 3h after HIE compared to the control group, identified by Lipid maps database.

p-value	Adduct	FC	Name	m/z
0.002955	+[M+Na]	2.98	FA 10:1	193.128
0.001321	+[M+H]	3.9	NAE 10:2	212.156
0.0048154	+[M+H]	3.56	FA 14:0	229.21
0.0047116	+[M+H]	2.44-	FA 16:0	257.254
0.001677	+[M+H]	4.15	MG 12:3	269.181
0.0027888	+[M+Na]	2.61	SPB 16:3;O	274.223

0.00000047758	+[M+Na]	2.92	FA 16:1	277.223
0.0019779	+[M+H]	6.87	MG 14:4	295.199
0.0049614	+[M+H]	6.5	MG 14:3	297.211
0.00036712	+[M+Na]	2.51	NAE 14:4;O	302.173
0.0021887	+[M+H]	29.88	MG 14:0	303.261
0.00014326	+[M+Na]	2.14	FA 20:5	325.208
0.0039525	+[M+H]	5.37-	NAE 18:4;O2	352.255
0.0017235	+[M+H]	19.05-	NAE 20:1	354.344
0.0047674	+[M+H]	2.77-	FA 22:0;O	357.328
0.0033678	+[M+H]	3.41	CAR 14:2;O3	416.254
0.00050525	+[M+H]	8.12	LPE 16:4	446.233
0.0028741	+[M+H]	4.34	CAR 20:4	448.346
0.0046577	+[M+H]	3.61	LPE 16:2	450.255
0.0021678	+[M+H]	26.48	DG 24:5;O	463.298
0.0049951	+[M+H]	2.32	LPI O-14:0;O	547.282
0.00015242	+[M+H]	2.53	CerP 30:6;O2	552.342
0.0028927	+[M+H]	3.68	DG O-30:0;O2	559.495
0.0016023	+[M+Na]	2.24	PA O-24:0;O	561.351
0.0037796	+[M+Na]	2.34	CAR 22:4;O4	562.332
0.00044709	+[M+H]	3.56	LPT 20:1	566.338
0.0040089	+[M+Na]	3.11	Cer 36:6;O2	578.462
0.0009415	+[M+Na]	2.67	DGTA 22:6	582.33
0.0022787	+[M+H]	2.43	ST 22:1;O5;HexNAc	584.348
0.00026896	+[M+Na]	4.85	PA 26:1	585.352
0.0024853	+[M+Na]	2.44	DG O-34:8	589.425
0.0021688	+[M+H]	5.31-	DG 32:0;O2	601.499
0.0048164	+[M+H]	2.49	CAR 28:7;O4	618.396
0.0012356	+[M+Na]	3.67-	SM 28:6;O3	647.381
0.0007614	+[M+Na]	4.07	PC O-30:4	706.479
0.0025135	+[M+H]	21.88	HexCer 38:5;O4	780.566
0.000000046454	+[M+H]	4.51	CerP 44:0;O4	792.65
0.0048854	+[M+Na]	3.14	DG 48:7	801.638
0.0038792	+[M+H]	2.59	TG 48:3	801.699
0.0034685	+[M+Na]	2.54	Cer 52:6;O2	802.704
0.0004365	+[M+Na]	6.18	Cer 52:5;O2	804.722
0.000011383	+[M+Na]	2.4	PA 42:2	807.59
0.0015535	+[M+H]	3.67	HexCer 38:4;O6	814.574
0.0043849	+[M+H]	4.5	PE 42:8	816.558
0.00000082469	+[M+H]	4.05	PC 38:0	818.655
0.00000078533	+[M+H]	3.31	CerP 46:0;O4	820.669
0.0035749	+[M+H]	3.3	HexCer 40:2;O5	830.641
0.0025924	+[M+H]	3.77	Cer 54:0;O3	836.852
0.000085167	+[M+H]	4.12	PC O-42:6	848.654
0.0031285	+[M+H]	4.41	Cer 54:6;O5	856.744

0.0034666	+[M+H]	18.42	DG 52:0;O	865.825
0.0010949	+[M+H]	2.87	DG O-52:0;O2	867.831
0.0010307	+[M+H]	3.29	PE O-46:3	868.71
0.0033345	+[M+H]	5.8-	IPC 40:4;O3	874.577
0.0030906	+[M+H]	5.94	CE 34:4;O	885.81
0.00030184	+[M+Na]	4.24	PE 46:3	904.666
0.0040205	+[M+H]	2.59	PE O-50:5	920.749
0.00079384	+[M+H]	2.14	ACer 58:0;O4	922.89
0.000016214	+[M+H]	5.82	PC 46:11;O	924.62
0.00095545	+[M+H]	3.23	ACer 60:5;O3	924.846
0.0013647	+[M+H]	2.77-	SM 48:3;O4	927.763
0.00014313	+[M+H]	2.11	PC 46:1;O	944.774
0.00089473	+[M+H]	5.68	TG 62:13	977.765
0.0040545	+[M+H]	3.12	SM 52:0;O4	989.873
0.00039065	+[M+H]	4.62-	HexCer 52:0;O6	1018.842
0.00083671	+[M+H]	2.43	PC 52:4;O	1022.825

Table S2. Possible biomarkers with statistically significant changes in concentration at 6h after HIE compared to the control group, identified by Lipid maps database.

p-value	Adduct	FC	Name	m/z
0.0016416	+[M+Na]	2.89	FA 10:1	193.1282
0.0014857	+[M+Na]	2.45	FA 12:3;O2	249.1197
0.0039697	+[M+Na]	6.1	MG O-14:3	305.2155
0.0012847	+[M+H]	2.56	NAT 12:3;O	318.1477
0.0013296	+[M+H]	2.72	MG O-18:3	339.2986
0.0015763	+[M+H]	6.45-	NAE 20:1	354.3444
0.00072378	+[M+Na]	3.13-	FA 22:4	355.2706
0.0037989	+[M+Na]	3.84	ST 24:1;O8	479.2538
0.0046382	+[M+H]	3.18	DG 28:4;O	521.3805
0.0043514	+[M+H]	4.04	Cer 40:6;O2	612.545
0.0045998	+[M+H]	2.23	Cer 42:2;O	632.6268
0.0023906	+[M+Na]	4.09	PS 24:4	638.3132
0.0019621	+[M+Na]	4.45	PS 26:4;O	682.3403
0.00021827	+[M+Na]	11.49-	SM 34:5;O2	717.499
0.0002511	+[M+NH4]	2.74-	PC 34:0	779.6351
0.0014723	+[M+H]	19.08	HexCer 38:5;O4	780.5663
0.0029146	+[M+H]	4.39	CerP 42:3;O6	790.557
0.00011604	+[M+H]	3.49	CerP 44:0;O4	792.6495
0.00030218	+[M+Na]	8.73	Cer 52:5;O2	804.7221
0.0010706	+[M+H]	8.53	DG 48:0;O	809.7557
0.00094651	+[M+H]	2.3	CerP 46:0;O4	820.6694
0.0039928	+[M+H]	3.94	TG 56:15;O2	921.6142
0.00013788	+[M+H]	2.2	PC 46:1;O	944.7742
0.0034829	+[M+Na]	2.69	PC 46:0;O	968.775

0.0035041	+[M+Na]	7.5	CerPE 52:1;O5	983.7665
0.0022335	+[M+H]	2.55	PC O-50:0;O	988.8665

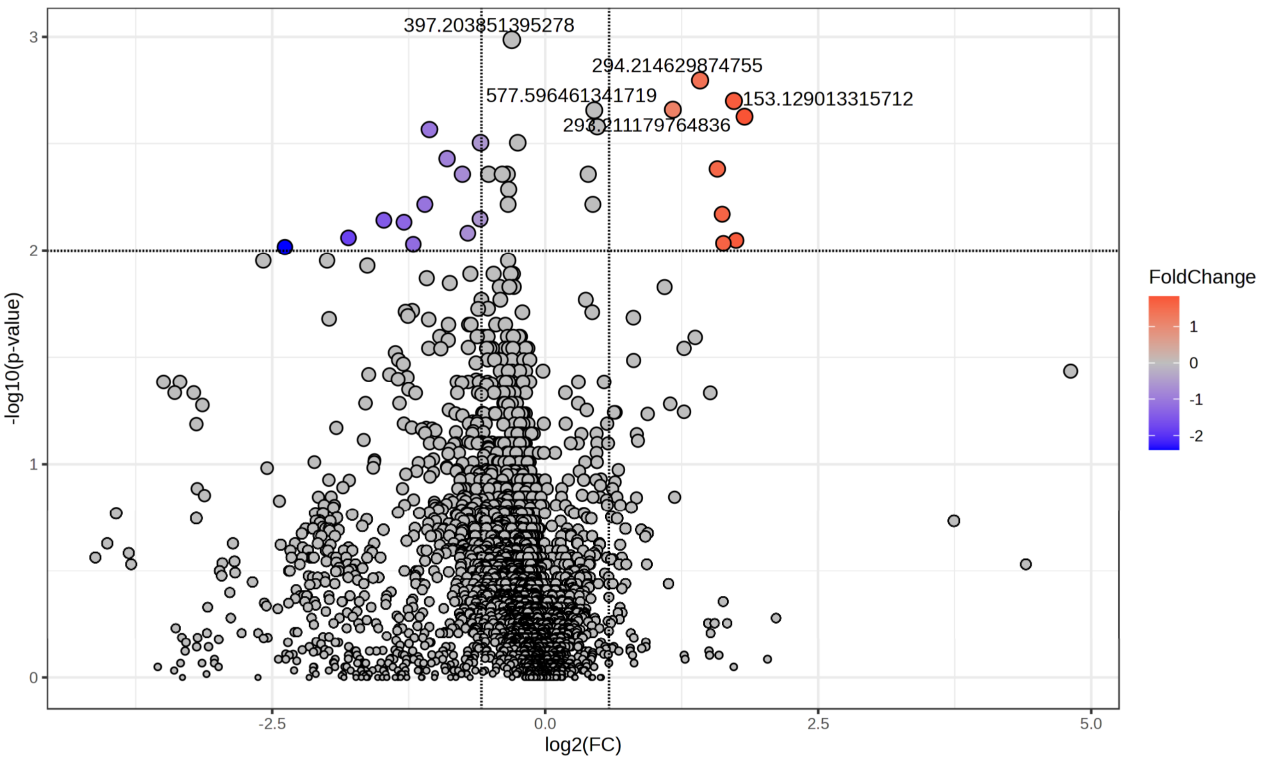

Figure S3. Volcanoplot of DBS metablolites on HIE-hypothermia group compared to HIE-Normothermia.

Table S3. Possible biomarkers with statistically significant changes in concentration in hypothermia group compared to the normothermia group, identified by LIPID MAPS database

m/z	Name	FC	Ion	P-val
261.1981	FA 14:0;O2	-2.28	[M+H]+	0.046551
293.2112	FA 16:1;O	2.25	[M+Na]+	0.0021857
294.2146	FA 14:0;O3	2.67	[M+NH4]+	0.0015955
337.2376	FA 18:1;O2	2.98	[M+Na]+	0.0041549
338.241	CAR 12:3	3.1	[M+H]+	0.0092367
404.3584	NAE 24:4	-2.1	[M+H]+	0.02106
424.2855	LPC O-12:1	-2.09	[M+H]+	0.0027144
463.4239	ST 30:0;O3	28.09	[M+H]+	0.03669
537.1697	ST 24:4;O7;S	-10.5	[M+Na]+	0.046394
538.3909	LPE 22:0	2.41	[M+H]+	0.028718
538.3909	NAE 30:6;O2	2.41	[M+Na]+	0.028718
584.2744	LPI 16:3	-2.12	[M+NH4]+	0.013511
606.6248	Cer 40:1;O	2.13	[M+H]+	0.014862
633.5114	Cer 38:6;O4	-2.45	[M+NH4]+	0.007368

644.4015	PE 28:4;O	-2.78	[M+H]+	0.0072106
661.5066	CAR 32:6;O2	-2.59	[M+NH4]+	0.030118
670.4491	PC 28:4	-2.39	[M+H]+	0.02028
709.4023	PG 32:7	-2.33	[M+H]+	0.019178
737.5845	SM 34:0;O4	-2.55	[M+H]+	0.0401
790.631	DG 48:10	-2.31	[M+NH4]+	0.0093317
796.5131	PT 36:5	-2.43	[M+H]+	0.019359
830.7069	HexCer 42:0;O3	-3.49	[M+H]+	0.0087136
845.7303	ACer 52:5;O4	-2.09	[M+NH4]+	0.028718
850.7179	TG 48:3;O2	-3.07	[M+NH4]+	0.038056
862.7547	TG 50:3;O	-2.46	[M+NH4]+	0.033992
880.7634	TG 50:2;O2	-3.09	[M+NH4]+	0.011787
934.7588	TG 56:9;O	-2.69	[M+NH4]+	0.038151
946.8489	TG 56:3;O	-2.4	[M+NH4]+	0.039303
1186.0537	PC 62:1;O	-2.54	[M+NH4]+	0.032503

Figure S4 Most enriched pathways in hypothermia-normothermia comparison according to integrated KEGG-Reactome-WikiPathways database (RaMP-DB)

Metabolite Set	Total	Hits	Expect	P value	Holm P	FDR
Arachidonic acid (AA, ARA) oxylipin metabolism	78	12	0.437	1.27E-15	4.21E-12	4.21E-12
Eicosanoid metabolism via lipooxygenases (LOX)	32	7	0.179	2.05E-10	6.8E-7	3.4E-7
Leukotriene C4 Synthesis Deficiency	60	7	0.336	2.13E-8	7.07E-5	2.15E-6
Rofecoxib Action Pathway	60	7	0.336	2.13E-8	7.07E-5	2.15E-6
Salicylate-sodium Action Pathway	60	7	0.336	2.13E-8	7.07E-5	2.15E-6

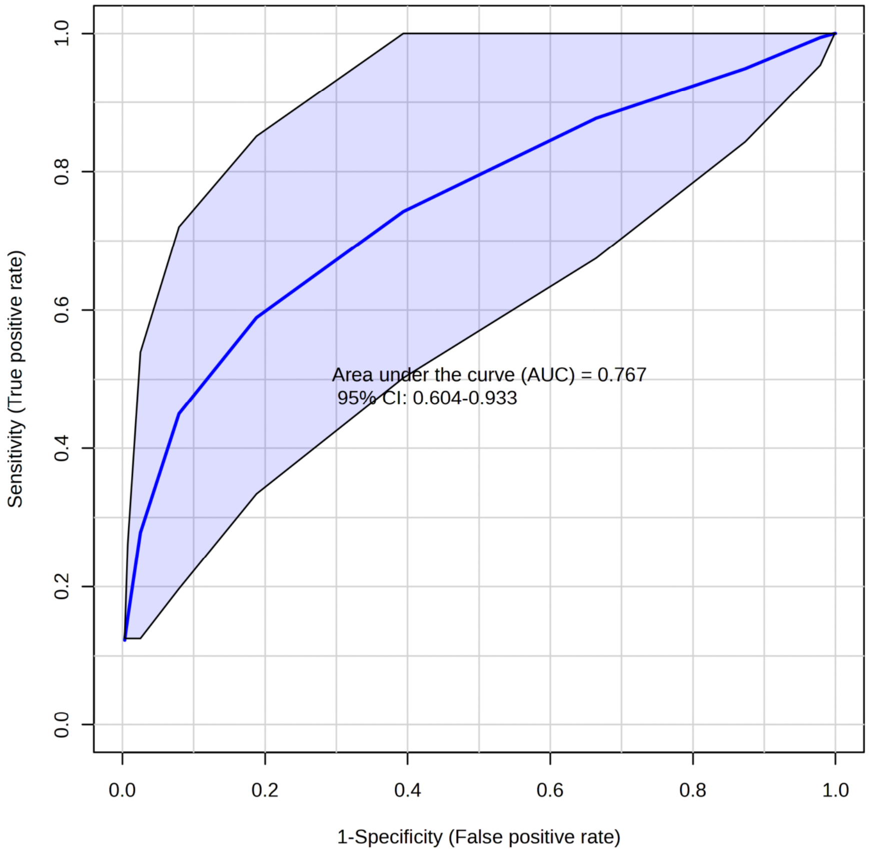

Figure S5. ROC curve for Intact HIE-6h group based on OPLS-DA model.
